# Lack of sex‐specific movement patterns in an alien species at its invasion front – consequences for invasion speed

**DOI:** 10.1002/ece3.2300

**Published:** 2016-07-14

**Authors:** Ivar Herfindal, Claudia Melis, Per‐Arne Åhlén, Fredrik Dahl

**Affiliations:** ^1^ Department of BiologyCentre for Biodiversity DynamicsNorwegian University for Science and TechnologyN‐7491TrondheimNorway; ^2^ Swedish Association for Hunting and Wildlife ManagementÖster MalmaSE‐61191NyköpingSweden; ^3^ Department of EcologySwedish University of Agricultural SciencesGrimsö Wildlife Research StationSE‐73091RiddarhyttanSweden

**Keywords:** Dispersal, invasive species, movement ecology, *Nyctereutes procyonoides*, raccoon dog

## Abstract

Efficient targeting of actions to reduce the spread of invasive alien species relies on understanding the spatial, temporal, and individual variation of movement, in particular related to dispersal. Such patterns may differ between individuals at the invasion front compared to individuals in established and dense populations due to differences in environmental and ecological conditions such as abundance of conspecifics or sex‐specific dispersal affecting the encounter rate of potential mates. We assessed seasonal and diurnal variation in movement pattern (step length and turning angle) of adult male and female raccoon dog at their invasion front in northern Sweden using data from Global Positioning System (GPS)‐marked adult individuals and assessed whether male and female raccoon dog differed in their movement behavior. There were few consistent sex differences in movement. The rate of dispersal was rather similar over the months, suggesting that both male and female raccoon dog disperse during most of the year, but with higher speed during spring and summer. There were diurnal movement patterns in both sexes with more directional and faster movement during the dark hours. However, the short summer nights may limit such movement patterns, and long‐distance displacement was best explained by fine‐scale movement patterns from 18:00 to 05:00, rather than by movement patterns only from twilight and night. Simulation of dispersing raccoon dogs suggested a higher frequency of male–female encounters that were further away from the source population for the empirical data compared to a scenario with sex differences in movement pattern. The lack of sex differences in movement pattern at the invasion front results in an increased likelihood for reproductive events far from the source population. Animals outside the source population should be considered potential reproducing individuals, and a high effort to capture such individuals is needed throughout the year to prevent further spread.

## Introduction

Threats from invasive alien species on ecosystems have received increased attention and resulted in national and international actions plans and conventions aiming at reducing the spread and establishment of alien species (e.g., European Parliament and Council 2014). Management actions to reduce the spread of alien species should be based on a three‐step approach: prevent, control, and eradicate (Secretariat of the Convention on Biological Diversity 2001). Actions to reduce further spread of alien species require knowledge about how they move in a novel environment and under different ecological conditions than those found in established populations for instance in the natural range of the species (Rout et al. 2014).

Individuals at the invasion front face some important differences compared to individuals in established populations, which may weaken the applicability of results from studies on the latter to predict movement of the former. Environmental factors such as habitat and climate may differ. These factors are detrimental for the costs and benefits associated with movement and, accordingly, for expected movement of individuals. Moreover, ecological factors such as population abundance, age structure, and sex structure may differ from those found in established populations, further affecting individual variation in dispersal patterns, for instance between males and females (e.g., Bowler and Benton 2005; Mabry et al. 2013). This has several important implications. Finding a mate becomes more challenging at the invasion front because the probability to encounter conspecifics is low at low population abundances, resulting in dispersal to be longer in both time and distance. If one individual in an established pair dies, this may force the mate to start dispersing because the likelihood to find an “available” partner nearby is low. This is particularly relevant because the eradication effort of invasive alien species is often high at the invasion front, resulting in that one individual in a pair is killed. These factors may for instance lead to a higher proportion of dispersing adults at the invasion front compared to what is reported from established populations. Age‐ or stage‐specific dispersal patterns (e.g., Bowler and Benton 2009) imply that studies from established populations with low proportion of adult dispersers not necessarily can be used as a basis for the management of invasive populations.

The speed of range expansion is related to factors such as dispersal rate (i.e., the proportion of a cohort that disperses), the dispersal speed and distance, and the likelihood to establish and reproduce in new areas (Davidson et al. 2011; Sih et al. 2012). Range expansion may also be related to individual differences in dispersal behavior. For instance, sex‐specific dispersal may affect the encounter rate between males and females, with consequences for settlement and reproduction in new areas (Lapointe et al. 2013). In mammals, males often have higher dispersal rate (Swenson et al. 1998) and longer dispersal distance (Favre et al. 1997; Nagy et al. 2013). Such sex‐specific dispersal can give an invasion front dominated by males, resulting in few mate encounters and a slower invasion rate compared to species with similar dispersal behavior in males and females (Swenson et al. 1998; Andersen et al. 2004). Predicting the spatial and temporal scale of range expansion therefore requires knowledge about sex‐specific variation in movement pattern. Moreover, animal movement is related to factors such as heterogeneity and predictability of the environment which varies temporarily (e.g., McIntyre and Wiens 1999; van Moorter et al. 2013). These factors may have different consequences for the short‐ and long‐term movement patterns, and it is therefore important to understand how short‐term spatial behavior translates into long‐term displacement for invasive species.

The raccoon dog is a medium‐sized canid native to eastern Asia and has through several introduction events between 1929 and 1955 in the former Soviet Union established viable and abundant populations in Europe (Kauhala and Kowalczyk 2011). In the period from 1935 to 1984, the raccoon dog colonized 1.4 million km^2^ of Europe by secondary expansion (Nowak 1984). Many countries have classified the species as an invasive alien species that should be eradicated (e.g., The Norwegian Directorate for Nature Management 2008; Directorate of Culture and Cultural and Natural Heritage 2009), following risk for spread of parasites and diseases (Ward and Wurster‐Hill 1990; Sutor et al. 2014) as well as concerns for negative impact on local ecosystems such as waterfowl colonies, although the scientific evidence for such negative ecosystem effects is not clear (e.g., Kauhala 1996b; Kauhala and Auniola 2001; Sutor et al. 2010). To follow‐up this commitment, a project was established in northern Sweden where raccoon dog has established a small reproducing population following invasions from viable and abundant populations in Finland (Kauhala and Kowalczyk 2011). The project should initially primarily monitor the established raccoon dogs and detect any new invaders and gather knowledge about the Swedish raccoon dog population, its behavior, and movement, in order to better target management actions (Dahl et al. 2010). Here, we use data from GPS‐marked individuals from this project to investigate raccoon dog movement at its range expansion front, with particular emphasis on temporal variation and sex differences.

The raccoon dog is a highly monogamous species. Once sexually mature, a raccoon dog start dispersing to find a partner. When a pair has been formed, they settle in a confined home range where they breed and stay year‐round (Kauhala et al. 1993). When one of a pair dies, the remaining partner once again starts searching for a new partner (Dahl et al. 2010). This behavior is used in the management of the species (Dahl et al. 2010). Captured raccoon dogs are fitted with a GPS/satellite transmitter and tracked remotely to find new raccoon dogs through the transmitter animal. When the raccoon dog settles in a restricted area, it is visited physically to search for a partner using specially trained dogs. The dogs usually, but not always, find the partner if there is one. If no partner is found at the first visit, the transmitter animal is revisited regularly as long as it stays in the same area or a partner is found. A captured pair is usually split up, and one of the partners is moved to another area to search for a new partner. If the number of transmitter animals is high enough, new animals are culled as part of the management.

To avoid unwanted reproductions, if the transmitter animals are lost, all captured raccoon dogs are sterilized by vasectomy for males and by tubal ligation for females (Dahl et al. 2010) before the release back in the nature. The sterilization procedure retains all hormonal activity of the individual, but any mating attempt will be nonsuccessful as the individuals are not fertile. Such sterilization methods have been shown to not affect natural behavior in other animals (Bromley and Gese 2001; Seidler and Gese 2012), except of course during the period of raising offspring for reproducing individuals. It is important to emphasize that the individuals are sterilized, but not neutered which would probably have affected their drift to find a partner. Accordingly, activities, such as mate search, pair bounding, and mating, is expected to be similar as for unsterilized individuals, but there will be no cost of reproduction associated with producing and rearing offspring. The management in itself is however affecting the structure of the population. Since we split up pairs when found, there is likely a higher proportion of single animals searching for a partner in our population than what would have been the case if it was not managed. Our general goal with this study is to assess the dispersal potential at the invasion front, that is, where the population density is very low and mate search often is unsuccessful. Our managed population may be viewed as an extreme population, at the very edge of the expansion front, and show the potential behavior and dispersal patterns during such circumstances. This implies that we can study natural movement behavior of individuals that do not reproduce, that is, a common situation for individuals at the invasion front where potential mates are scarce. Accordingly, our study can add valuable information regarding behavior of invasive species in areas where such information is most crucial to obtain (i.e., at the invasion front), but caution should be made regarding generalization of our results to abundant and established populations. See Methods and Supporting information for more details regarding animal handling and consequences for natural behavior.

Temporal pattern in raccoon dog movement is shaped by long‐term (seasonal) and short‐term (diurnal) variation in food abundance and mortality risk (Sutor and Schwarz 2013; Melis et al. 2015). In northern regions where winter conditions are harsh, raccoon dogs normally adopt winter sleep (lethargy) by decreasing metabolism by about 25% (Asikainen et al. 2002). The winter survival and next spring body condition depend mainly on fat reserves gained during summer and autumn (Kauhala 1993), when adult raccoon dogs can almost double their weight (Kauhala 1996a; Melis et al. 2010). Being an omnivore, there are no clear diurnal patterns in food availability for raccoon dogs. However, the costs of movement may still vary diurnally, for instance according to mortality risk by predators or humans (Drygala et al. 2010). Raccoon dogs form permanent pairs long before the breeding season and male and female share territory (Kauhala et al. 1993, 1998). Combined with the lack of sexual size dimorphism (Kauhala 1993), few sex differences in space use pattern and habitat utilization have been detected (Kauhala et al. 1993; Melis et al. 2015), even if there are some differences in movement pattern during the denning period (Drygala et al. 2008b). There is also little evidence for any overall difference between male and female in dispersal behavior such as dispersal distance (Drygala et al. 2010).

Much of our knowledge about raccoon dog outside it native area comes from established high‐density populations, for instance in southern Finland and central Europe (e.g., Kauhala et al. 2007; Sutor 2008; Kowalczyk et al. 2009). Studies from the invasion front are rarer (but see Drygala et al. 2010) and often based on few individuals or low‐resolution data (e.g., radio‐telemetry or visual observation of marked individuals). Here, we take advantage of the high number of GPS‐marked raccoon dog in northern Sweden, the invasion front of the large and abundant Finnish population (Kauhala and Kowalczyk 2011), to address sexual and temporal patterns in movement behavior at the invasion front. We had few *a prior* predictions regarding movement behavior at the invasion front. Our baseline expectations would thus be few sex differences but large long‐ and short‐term temporal variation in movement behavior as described above. We expect that individuals will show strong dispersal behavior due to the low abundance of conspecifics and potential mates. This could affect both the temporal pattern (e.g., if the trade‐off between risk avoidance and search for conspecifics/mate is weakened) as well as introduce sex differences for instance if one sex is more risk‐adverse compared to the other or due to inbreeding avoidance mechanisms (e.g., Bowler and Benton 2005; Mabry et al. 2013; Kamler and Macdonald 2014). We also estimated dispersal distances in relation to movement characteristics and finally assessed how the lack of sex‐specific movement pattern affected the encounter probability and distance kernel of encounters between males and females.

## Methods

### Raccoon dog data

As part of a management project (The Swedish Raccoon dog project, NV‐ Dnr 802‐0289‐08, LIFE09 NAT/SE/000344), 55 raccoon dogs were captured. The captured individuals were sterilized, aged, and sexed and fitted with an ear tag and a GPS‐collar that was programmed to record a location every third hour, before released. For details regarding animal capture and handling and the study area, see Melis et al. (2015). During late autumn and winter, it is difficult to distinguish between adult and juvenile animals (Kauhala and Helle 1990; Kauhala 1993). Animals were all initially aged by weight and when carcasses from naturally dead transmitter animals were recovered also by incremental lines in the tooth cementum. Animals captured between March and September and weighing over 6 kg were all classified as adults (Kauhala 1993). Animals captured in October–December were classified as adults if they were weighing over the mean adult weight according to Kauhala (1993). Among the marked individuals, there were three likely juveniles, which were removed from further analyses as the sample size was considered too low to obtain reliable estimates of behavior of juveniles. In general, there were few possible juveniles in the sample as all obvious juveniles were culled for management reasons (transmitters were too heavy and we wanted adults for Judas animals) and those few we may have misjudged were too few to affect the general results of the study. The majority of the sample was captured in late spring until early autumn when juveniles are easy to distinguish from adults. Another five animals were only classified as adults by weight in late autumn; however, these animals were too few to affect the general results of the study even if all of them would have been juveniles. We also restricted data to individuals with observations for at least 20 days. One adult was removed due to suspected collar error, leaving a total of 21 males and 21 females for further analyses. Some transmitters were reprogrammed to obtain data on a higher temporal resolution, but in order to keep a standardized sampling protocol, we subsampled all data to 3‐h interval.

To ensure us that any patterns from our statistical models were not a result of our treatment of the animals (i.e., the sterilization and translocation of animals), we compiled some behavioral data from the GPS‐marked individuals. During the course of the study, 24 of our 55 initial study animals found at least one other raccoon dog, suggesting that sterilized animals search for and find partners, and establish pairs. Five of the formed pairs were kept intact for reference purposes. All pairs stayed together until one of the partners died or was moved by us (77–710 days), indicating that, even though they do not give birth to cubs, the pairs are still monogamous and stay together in restricted home ranges (mean = 10.84 and 10.84 km^2^ for males and females, respectively, for the first 50 days after capture and release, see Data S1, Fig. S1). Seven initially captured and unmoved raccoon dogs (three females and four males) that had found a partner were rereleased in the same place while the partner was culled or translocated, thus leaving the initial raccoon dog alone. Eight captured raccoon dogs (five females and three males) were translocated to another area and released. The data for both groups of animals stretch for as long as we could not confirm any new partner or the animal was lost or moved (two individuals were captured and released more than once). The unmoved and translocated animals showed similar displacement patterns (see Data S1, Fig. S2), indicating that translocated and unmoved individuals were similar with respect to propensity to move and search for a new partner after the treatment (i.e., removal of partner, or releasing into a novel area).

The trajectories were screened for observation error following Bjørneraas et al. (2010). We calculated movement speed as the distance between two locations (i.e., length of a step), and the turning angle as the change in direction from the previous step. Turning angles close to 0° mean that a step is in the same direction than the previous step, whereas values close to 180° or −180° mean that the individual moves back along the previous step (positive value is turn to the right, negative value is turn to the left). We used the absolute turning angle (henceforth: abs(turning angle)) that does not consider left or right turns in further analyses. We calculated daily locations using the daily center coordinate. The daily trajectories were used to calculate daily movement patterns (i.e., daily step lengths and abs(turning angle)), similar as for the 3‐h data.

We assigned information about solar elevation to the 3‐h data using the R (R Core Team 2015) package maptools (Bivand and Lewin‐Koh 2014). We used the following definition of photoperiod: solar elevation <−6°: night, solar elevation >−6° and <0°: dusk (before midnight) or dawn (after midnight), and solar elevation >0°: Day.

### Defining movement states

We used movement characteristics of daily steps to define behavioral state. We followed the approach described by van Moorter et al. (2010), with daily step length and abs(turning angle) as input movement characteristics (see also Melis et al. 2015). The method uses K‐means statistics to generate clusters, and a bootstrap procedure to assess the most likely number of clusters supported by the data (van Moorter et al. 2010). Based on gap statistics (cf. van Moorter et al. 2010), the procedure identified three clusters. The first cluster was recognized by long steps and low abs(turning angle), which would lead to directional movement and thus interpreted as dispersal state. The second and third clusters had shorter daily step lengths, but higher abs(turning angle). Because the biological meaning of cluster two and three appears similar, individuals not showing directional movement, but active, we pooled these two cluster into one class which we called “settled state.”

The clustering procedure did not account for temporal patterns, and each step was given a movement state independent of previous and following steps. We considered dispersal to be a behavioral state that persists for more than 1 day and that short periods of nondirectional movements in‐between longer periods of long‐distance movement were part of the dispersal state. To account for this, each step was assigned the behavioral state that was dominating during a 7‐day window around the step. If no state was dominating during the 7‐day window (i.e., the 7‐day period had no consistent movement pattern), the step was classified as nondirectional. As a consequence, some short steps or steps with high turning angle were assigned to the dispersal state because the majority of preceding or following steps had dispersal characteristics, and similarly, some long and directional steps were assigned to the nondirectional state. After the procedure, 11.8% of the daily steps were classified as dispersal steps with a mean step length of 4147 m and an absolute turning angle of 64.7°, suggesting a movement away from previous locations. The remaining 88.2% was classified as nondirectional movement state and had a mean step length of 1295 m and absolute turning angle of 109.3°, indicating movement toward previous steps.

The movement state of an individual in a given day was then assigned to all 3‐h locations for the individual that day.

### Statistical analyses

The classification of movement state treated all steps similarly, and it did thus not provide any information about temporal or sex‐specific patterns, which we were particularly interested in. Accordingly, to assess the extent of such patterns, we evaluated movement characteristics (i.e., step length and turning angle) with respect to season, time of day, and sex, even if the movement states are defined by the same movement characteristics. This was possible because short‐ or long‐term temporal patterns in addition to sex are not included in the definition of movement states.

In all statistical models, we included the trajectory identity (i.e., the combination of animal identity and recapture number of the individual) as a random factor to account for interdependency between observations belonging to the same trajectory (e.g., Bolker et al. 2009). Step length was ln‐transformed in all analyses to reduce heteroscedasticity.

Movement patterns are expected to vary in time in a continuous manner. We therefore used generalized linear additive models (gamm; Wood 2006) to model the temporal variation in raccoon dog movement. This category of models uses regression splines to fit nonlinear relationships in the data and is particular useful for assessing nonlinear patterns when an a priori relationship cannot be defined, such as many ecological patterns involving time as a covariate (Zuur et al. 2009). We used cyclic cubic splines with maximum number of knots set to six to account for the periodicity of both the annual and diurnal patterns. As we were particularly interested in whether males and females differed in movement behavior, we included sex as an interaction with the cyclic cubic splines. Movement steps belonging to the dispersal and nondirectional states were analyzed in separate models. This was performed to avoid overparameterized models, and also because we primarily were not interested in differences in movement characteristics between the behavioral states, but rather to search for sex‐specific movement patterns as this may have consequences for the speed of invasion.

To estimate differences and associated uncertainties between males and females in the movement at a given behavioral state, we used bootstrapping (*n* = 1000). In each iteration, we refitted the model and generated predictions of sex‐specific temporal patterns in movement characteristics. We then used the mean value of all the 1000 bootstrap iterations as the predicted value and 2.5% and 97.5% percentiles as lower and upper boundaries for the 95% confidence interval. This procedure also allowed us to calculate differences between male and female movement characteristics at a given time period for each iteration. If the 95% confidence interval of the difference did not include zero, sex differences in movement characteristics were considered significant.

To assess temporal pattern of dispersal rate and test for sex differences, we first calculated the frequency of daily steps belonging to the dispersal rate per trajectory for each month. This frequency of days with dispersal in a given month was then related to sex and month in a gamm with binomial family and logit link, and month as a cyclic cubic spline in interaction with sex as the explanatory variable. Further, we related daily step length and abs(turning angle) to month and sex in a gamm with gaussian family. This was performed separately for steps belonging to the dispersal and nondirectional movement state.

Based on the results from the analyses at the daily movement steps, we choose to proceed with the 3‐h level analyses with months pooled to seasons (winter = December–March, spring = April and May, summer = June–August, autumn = September–November). We ran gamm on 3‐h movement characteristics (step length and abs(turning angle)) separately for each season and movement state. In each gamm, we modeled the diurnal pattern with cyclic cubic splines with an interaction with sex.

### The role of fine‐scale movement for large‐scale patterns

We assessed which fine‐scale movement characteristics (i.e., step length or turning angle from the 3‐h data) that best explained whether a daily step was classified as dispersal or nondirectional movement state. We did this to assess at what period of the day the movement best reflected the behavioral state, and whether it was turning angle or speed (step length) that best predicted raccoon dog dispersal. This was performed in a logistic regression with movement state (dispersal or nondirectional movement) as a binomial response variable and 3‐h step length and abs(turning angle) as explanatory variables. We ran separate models for each season and 3‐h interval. Step length and turning angle were standardized prior to modeling to be able to compare the parameter estimates between the two characteristics. Bootstrapping (*n* = 1000) was used to assess 95% confidence interval.

We then evaluated which movement characteristics best predicted long‐term movement as measured by displacement during a 50 days period. This was performed by calculating the distance between the first and fiftieth location for each individual trajectory (i.e., the net displacement). Some trajectories were shorter than 50 days, and we then calculated the expected displacement after 50 days by: (displacement/number of days)*50, however, excluding trajectories of less than 25 days. All trajectories were continuous, that is, not irrupted by capturing or handling. For each individual subset of trajectories, we calculated the mean abs(turning angle) and step length based on the 3‐h data. We then regressed the net displacement (ln‐transformed) against step length (ln‐transformed) and abs(turning angle). We also included the interaction between step length and abs(turning angle), as well as sex and its two‐ and three‐way interactions with the movement characteristics. We used AIC corrected for small sample size (AICc, Burnham and Anderson 2002) to evaluate the importance of variables in explaining individual variation in net displacement. AICc provides a measure of how parsimonious a model is compared to alternative candidate models, and models with difference in AICc (ΔAICc) less than two should be considered as equally supported by the data (Burnham and Anderson 2002). The procedure was run five times with different ways to calculate fine‐scale movement characteristics (i.e., step length and abs(turning angle)): (1) mean values of all 3‐h steps, (2) mean values from all steps during the dark hours based on photoperiod (i.e., steps during twilight and night), (3) mean values of steps from 18:00 to 5:00, (4) mean values from all steps during the light hours based on photoperiod (i.e., daytime), (5) mean values of steps from 6:00 to 17:00. We did this to assess whether raccoon dog trade‐off risk avoidance for long‐distance displacement by expanding dispersal activity into nondark hours in periods with very short nights such as mid‐summer.

### Modeling dispersal and male–female encounters

We then simulated spatial and temporal distribution of encounters between male and female dispersers by generating random walks of individuals starting at the same location. The purpose of the simulation was to get an impression of how the (lack of) sex‐specific movement patterns affected the encounter rates and distances relative to other scenarios for sex‐specific movement patterns, as well as an idea about at what distances from the core area encounters between male and female raccoon dog could take place, which could have implications for how invasion potentially can take place.

Ten males and ten females started at the same core area (represented by the same location) but with two time steps (days) between each pair, such that at each dispersal event, one male and one female started from the core area. The direction of the first step from the starting location was randomized for each individual. Each step was drawn from the daily steps based on the GPS‐data and belonging to the dispersal movement state, but varied for males and females in three different scenarios (Table 2): (1) similar movement of males and females (male and female steps were randomly drawn from the pooled male–female real steps), (2) long steps for males and short for females (male random steps were drawn from male real steps, female random steps were drawn from the steps belonging to the lower 50% percentile of the female real steps), (3) real data (male steps randomly drawn from male real steps, female steps randomly drawn from female real steps). A trajectory was allowed to continue for 50 days (steps) unless it had encountered a trajectory belonging to the opposite sex. The crossing steps from the two trajectories should also occur the same day, and if so, both trajectories were stopped and the crossing location was defined as an encounter between a male and a female. The distance from the starting location and days since start of dispersal were recorded. We ran 1000 simulations for each of the three scenarios.

## Results

The 6084 daily steps from 42 raccoon dogs with sufficient data to be included in the statistical analyses had an average daily step length of 1658 m (2298 SD) and an absolute turning angle of 103.9° (58.3 SD). For the 3‐h data, average step length was 496 m (787 SD) and abs(turning angle) was 96.5° (55.6 SD).

The longest displacement (i.e., distance from the release location) was a male that was released at the same location as it was captured May 20, 2010 and started moving north 2 weeks later with an extraordinary high speed. Hundred days after it was released it was located 360 km away. A few days later the GPS stopped working, but the individual was shot a few months later in the same area. Mean displacement among all individuals 100 days after release was 21.7 km (SD = 59.8).

### Seasonal movement patterns

**Figure 1:**
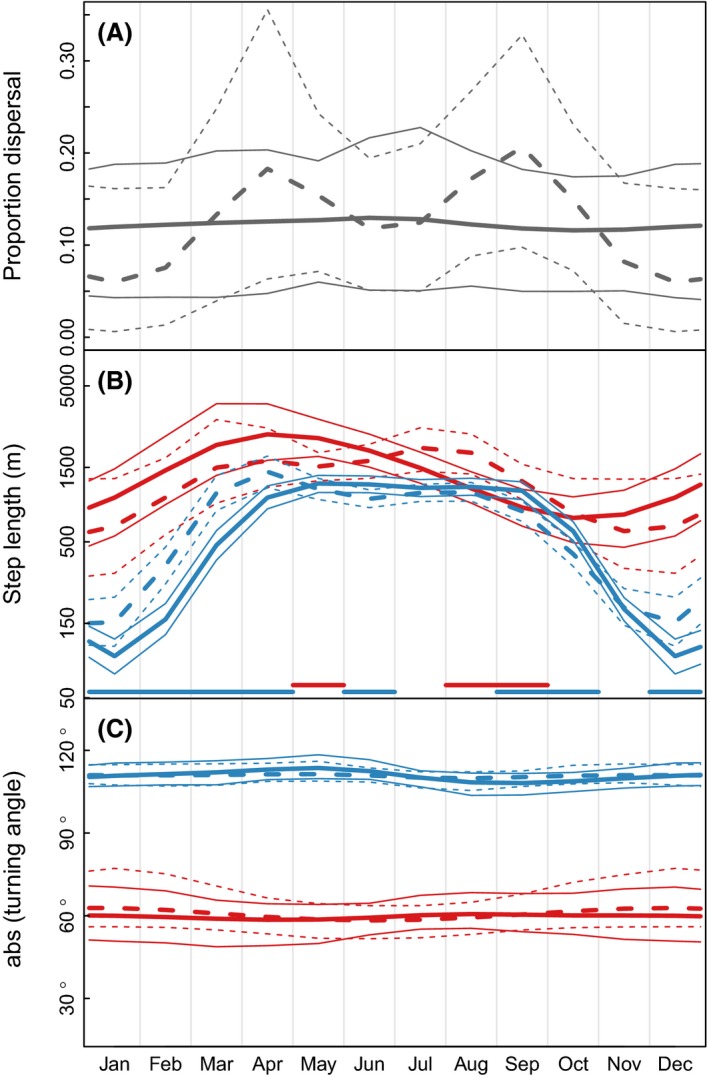
Monthly movement patterns for female (solid lines) and male (dashed lines) raccoon dog. In panels B and C, red and blue lines are temporal patterns for individuals at the dispersal and nondirectional movement state, respectively. Thin lines show 95% credible interval based on bootstrapping (*n* = 1000). Horizontal lines at the bottom of each panel indicate periods where movement patterns for females and males differ significantly, that is, 95% credible interval of the difference between male and female from bootstrapping (*n* = 1000) did not include zero.

There was little monthly variation in the proportion of steps belonging to the dispersal state (Fig. 1A). Indeed, the gamm suggested no temporal variation for either males or females, and the variation, that is, shown in Figure 1A is caused using mean from the bootstrapping as predicted temporal patterns. Still, the high uncertainty in the patterns as indicated by the 95% CI suggests that the rate of dispersal in raccoon dog is rather similar over the year. There was no evidence for differences in rate of dispersal behavior between males and females (95% CI of the difference between males and females included zero in all months).

Males had on average somewhat longer daily step lengths compared to females both at the dispersal state (males: mean = 4239 m, SD = 4350, females: mean = 2857 m, SD = 3212) and the nondirectional movement state (males: mean = 1503 m, SD = 1931, females: mean = 1214 m, SD = 1475). The gamm suggested that daily step length was longer during summer and shortest during winter (Fig. 1B). This pattern was clearer for individuals in the nondirectional movement state compared to individuals at the dispersing state. There was also evidence for some sex differences in the annual pattern in step length. At the dispersal state, significant sex difference in step length was found during May (females had longer steps than males) and in August and September (males had longer steps than females, Fig. 1B). At the nondirectional movement state, males had significantly longer daily steps compared to females from December to April, whereas females had longer steps than males in June, September, and October (Fig. 1B).

There was little difference in abs(turning angle) between males and females at the dispersal (males: mean = 59.42°, SD = 57.01, females: mean = 58.34°, SD = 57.96) and nondirectional movement state (males: mean = 111.08°, SD = 55.56, females: mean = 111.23°, SD = 54.86). Basically, no temporal variation was present in either of the behavioral states, and no significant sex differences were detected (Fig. 1C).

### Diurnal movement patterns

**Figure 2:**
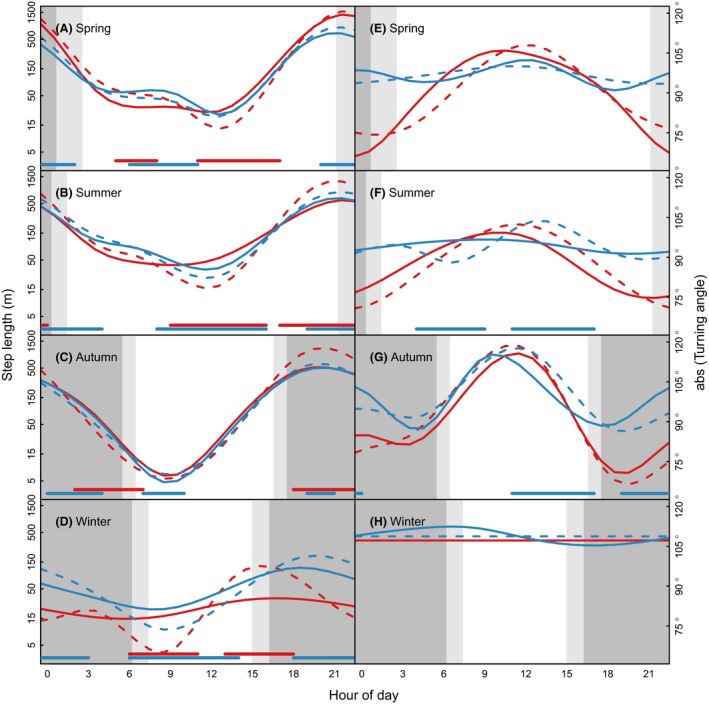
Seasonal and diurnal movement patterns (left panel = step length, right panel = turning angle) of female (solid lines) and male (dashed lines) raccoon dog at dispersal (red) or nondirectional (blue) movement state. Thin lines show 95% credible interval based on bootstrapping (*n* = 1000). Horizontal lines at the bottom of each panel indicate periods where movement patterns for females and males differ significantly, that is, 95% credible interval of the difference between male and female from bootstrapping (*n* = 1000) did not include zero. White, light gray and dark gray background indicates day, twilight, and night hours, respectively.

The 3‐h step lengths did not differ much between males and females when separating between steps from days at the dispersal state (males: mean = 664, SD = 1171, females: mean = 578, SD = 942) and nondirectional movement state (males: mean = 464, SD = 738, females: mean = 480, SD = 704). The gamm suggested large diurnal variation in step lengths, with longer steps during the dark hours and shorter steps during daytime (Fig. 2A–D). The diurnal pattern was weakest in winter (overall shorter step lengths throughout the 24‐h period) and strongest in autumn. The diurnal pattern in step lengths appeared similar for the dispersal and the nondirectional movement state. There was evidence for sex differences in step lengths during some periods. Most evident were: (1) In spring, dispersing males had significantly longer steps during early morning and shorter during afternoon compared to dispersing females (Fig. 2A). (2) In summer, females had significantly longer steps compared to males during mid‐day, irrespective of movement state, whereas males had longer steps during evening and night (Fig. 2B). (3) Although there was evidence for statistically significant different step length between males and females during autumn, this was very small and most likely not biologically significant (Fig. 2C). (4) The diurnal pattern in step lengths was weaker during winter, but there was some evidence for sex differences (Fig. 2D). Given the low frequency of locations per individual during winter (mean number of winter observation per individual = 12.9), these results should be interpreted with caution.

The turning angles for the 3‐h data did not differ much between males and females at the dispersal state (males: mean = 89.60, SD = 56.94, females: mean = 91.90, SD = 56.82) or the nondirectional movement state (males: mean = 98.18, SD = 55.41, females: mean = 97.13, SD = 55.21). There were strong diurnal patterns in abs(turning angle) for individuals at the dispersal state, with lower values (more directional movement) during the dark hours compared to mid‐day in all seasons except during winter (Fig. 2E–H). Such diurnal pattern was not evident for individuals at the nondirectional movement state, except for the autumn (Fig. 2G). There was no significant sex difference in the diurnal pattern of abs(turning angle) for individuals at the dispersal state (Fig. 2E–H). At the nondirectional movement state, males had significantly sharper turns (moving in the opposite direction from the previous step) compared to females during early afternoon in summer and autumn, whereas this was reversed during morning in summers and late evening in autumn (Fig. 2F,G).

### The role of fine‐scale movement for large‐scale patterns

**Figure 3:**
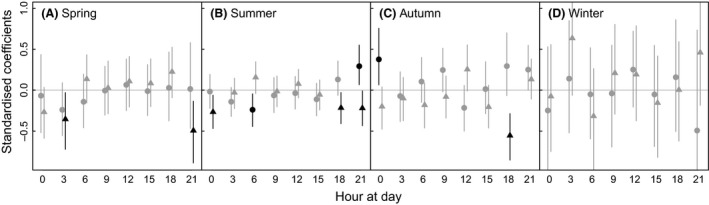
Standardized beta‐coefficients from logistic regression of the state of a daily step (dispersal or nondirectional movement) explained by fine‐scale (3‐h) step length (ln‐transformed) or absolute value of turning angle. Circles and triangles show coefficients for step length and turning angle, respectively. Positive values indicate that the probability that a daily step was a dispersal step was positively related to 3‐h step length or abs(turning angle) at the time of day the 3‐h step was from, indicated by the *x*‐axis. Bars represent 95% credible intervals based on bootstrapping (*n* = 1000); significant coefficients (95% CI did not overlap zero) are in black.

The probability that a daily step belonged to the dispersal state was best explained by fine‐scale variation in abs(turning angle) compared to fine‐scale variation in step length (six of the coefficients of abs(turning angle) were significant compared to three of the coefficients of step length; Fig. 3). For all seasons except winter, fine‐scale variation in turning angle significantly explained the probability that the daily step belonged to the dispersal state (Fig. 3). Fine‐scale step lengths significantly explained dispersal probability only during morning and night during summer (Fig. 3B) and at midnight during autumn (Fig. 3C). Note, however, that in summer the coefficient for step length at 6:00 was negative, suggesting that the probability that a daily step belonged to the dispersal state was higher if fine‐scale steps at 6:00 were short. Coefficients for winter were in general associated with very high uncertainties (Fig. 3D), most likely due to few individuals during this season.

**Figure 4:**
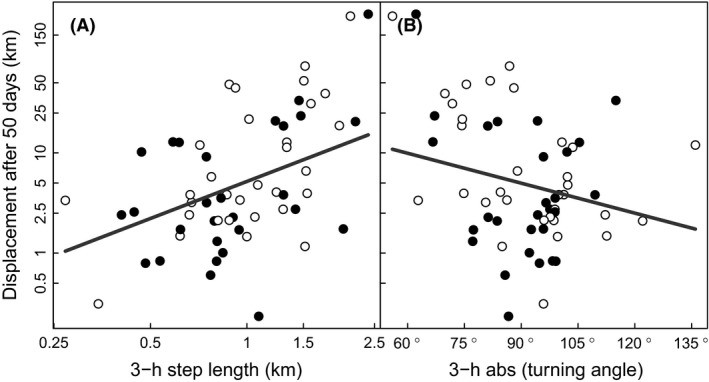
Relationship between fine‐scale movement patterns (A: step length, B: abs(turning angle), both measured at the 3‐h interval and from 18:00 to 05:00) and net displacement after 50 days for adult female (filled symbols) and male (open symbols) raccoon dogs. Step length and abs(turning angle) are measured as mean over the 50 days period. The lines show the predicted relationship from a log‐linear model (the model in bold from Table 1). Note the logarithmic *y*‐axis, and *x*‐axis in panel A.

Of the five methods to describe fine‐scale movement, the best explanatory power with respect to 50‐days displacement was achieved using steps from 18:00 to 05:00 (Table 1). This model suggested that net displacement was best explained by step length, abs(turning angle), and their interaction. The ΔAICc to the best model that included sex was 0.89, giving little support to sex‐specific displacement patterns. Net displacement increased with increasing step length and decreasing abs(turning angle) during night time (Fig. 4). The negative interaction suggested that the positive relationship between step length and net displacement was stronger when abs(turning angles) were low (i.e., a more directional movement).

**Table 1:** AICc‐based ranking of models explaining net displacement after 50 days from the start of a trajectory. Five different periods of the 24‐h period are used to calculate individual mean step length and abs(turning angle). The model in bold received most support from AICc‐values, and ΔAICc shows the difference in AICc between a candidate model and the model in bold. SL = step length (ln‐transformed) and TA = abs(turning angle)

Period	Sex	SL	TA	Sex*SL	Sex*TA	SL*TA	Sex*SL*TA	AICc	ΔAICc
All steps	X	X	X					204.46	1.27
	X	X						204.83	1.65
	X	X	X			X		204.93	1.75
		X	X			X		204.95	1.77
		X						205.07	1.88
Twilight + night		X	X					209.25	6.07
	X	X	X					209.76	6.58
		X	X			X		210.76	7.58
		X						211.18	8.00
	X	X	X		X			211.26	8.08
18:00–05:00		**X**	**X**			**X**		**203.18**	**0.00**
		X	X					203.52	0.34
	X	X	X			X		204.07	0.89
	X	X	X					204.34	1.16
		X						205.12	1.94
Daytime	X	X						212.12	8.94
		X	X			X		212.20	9.02
	X	X	X			X		212.81	9.63
		X						213.34	10.16
	X	X	X		X	X		213.35	10.17
05:00–18:00	X	X						216.07	12.88
		X						217.43	14.25
	X	X		X				218.21	15.03
	X	X	X					218.44	15.26
	X	X	X			X		219.06	15.88

### Long‐term displacement, encounter rates, and distances

**Figure 5:**
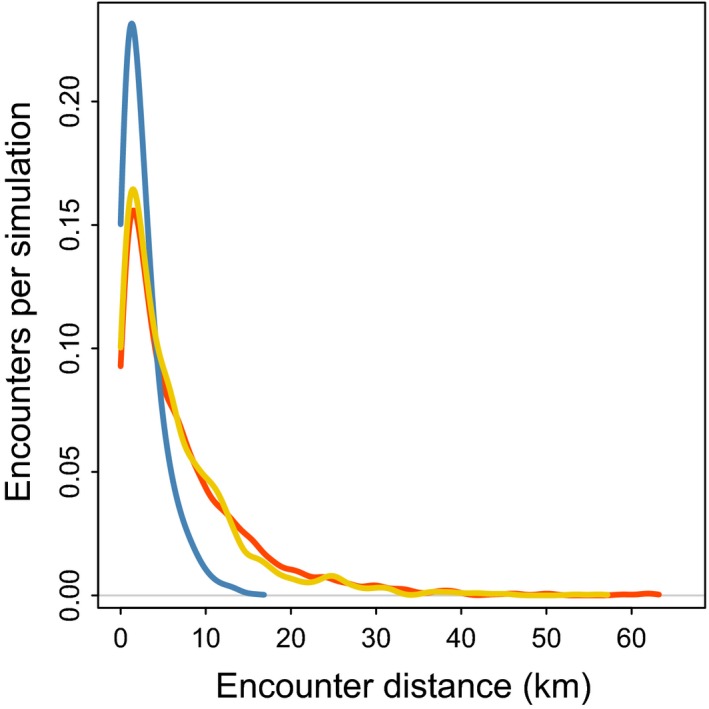
Probability of encounter between male and female raccoon dog in relation to distance, based on simulations from three movement scenarios: (1) males have long daily steps, female have short (blue line), (2) equal movement between males and females (both have long steps, red line), and (3) movement for males and females based on the empirical data (yellow line). See also Table 2.

Movement characteristics for male and female steps used in the three scenarios as well as summary statistics from the simulations are given in Table 2. The number of encounters per simulation and their distance distribution were similar for the “equal step” and “empirical data” scenarios, whereas the “male long, female short” scenario had lower frequency of encounter and shorter encounter distances than the two other scenarios (Table 2, Fig. 5). The number of days from start to encounter did not differ much between the three scenarios (Table 2).

**Table 2:** Summary statistics for distance between starting point and encounters between male and female raccoon dogs based on three scenarios: no sex differences in movement pattern, high male low female, and based on the empirical raccoon dog data. The first column summarizes the daily movement characteristics used to simulate individual trajectories (SL = step length (km), TA = turning angles (degrees)). Turning angles are given as absolute values in the table, but were added as real turning angles in the simulations. All distances are given in kilometers

Scenario	Movement characteristics	Number of encounters	Encounter distance	Encounter time (days)
Equal movement	Male and female: SL = 3.70 (SD = 4.00) TA = 59.0 (SD = 57.4)	Mean = 1.40 SD = 1.01	Mean = 7.21 Median = 4.70 SD = 7.85	Mean = 15.1 Median = 14 SD = 7.60
Male long, female short	Male: SL = 4.28 (SD = 4.37) TA = 59.4 (SD = 57.0) Female: SL = 1.07 (SD = 0.87) TA = 62.4 (SD = 59.09)	Mean = 1.08 SD = 0.95	Mean = 2.69 Median = 1.99 SD = 2.48	Mean = 14.6 Median = 14 SD = 7.10
Empirical data	Male: SL = 4.28 (SD = 4.37) TA = 59.4 (SD = 57.0) Female: SL = 2.88 (SD = 3.22) TA = 58.3 (SD = 58.0)	Mean = 1.29 SD = 0.97	Mean = 6.56 Median = 4.27 SD = 7.11	Mean = 14.9 Median = 15 SD = 7.31

## Discussion

The role of landscape and life history effects of invasion success of alien species is well documented (e.g., Bullock et al. 2002; Bowler and Benton 2005; Capellini et al. 2015). Our results suggest that behavioral patterns particularly related to the lack of sex‐specific dispersal pattern may be an important factor for the invasion success of raccoon dog in Europe. Any sex differences in movement behavior occurred only as subtle differences in the temporal patterns in step length and turning angle (Figs 1B and 2). Although some of these differences received statistical support, we believe they have minor biological relevance, as supported by the results regarding long‐term movement pattern and simulation of encounters (Figs 4 and 5).

A species invasion speed is inevitably linked to individual dispersal distances (Bullock et al. 2002; Lockwood et al. 2007). It is therefore important to understand behavioral mechanisms behind long‐distance displacement. Previous studies on raccoon dogs have documented large individual and temporal variation in movement characteristics such as movement speed (i.e., measures of displacement) and activity rates (e.g., Kauhala et al. 2007; Sutor 2008; Drygala et al. 2010). Our results suggest that the fine‐scale tortuosity, that is, the deviation of a movement from a straight line (e.g., Codling et al. 2008), is a vital component for understanding long‐distance displacement in raccoon dog. For instance, to what extent a step was classified as dispersal was more often explained by fine‐scale tortuosity (turning angle) compared to fine‐scale step length (Fig. 3), and the displacement after 50 days was best explained by a combination of tortuosity and step length (Fig. 4). Accordingly, a combination of speed and tortuosity should be used when assessing the invasive behavior of a species (Doerr and Doerr 2004).

We found only a weak seasonal variation in dispersal rate (Fig. 1A), suggesting that nonreproducing adult raccoon dog without a partner show dispersal behavior throughout the year, differently than what is found in other studies on raccoon dog populations (Drygala et al. 2010), or the general pattern among mammals (i.e., a highly seasonal dispersal pattern: Bowler and Benton 2005). However, adult and juvenile dispersal pattern may differ (Bowler and Benton 2009), for instance because dispersal may be condition dependent and adults have more resources for long‐distance movement also in unfavorable conditions, or because juvenile dispersal is initiated at the end of parental care (Bowler and Benton 2005). Therefore, the discrepancy between our results and previous studies on raccoon dog dispersal behavior may be caused by differences in age and reproductive status (i.e., a large proportion of the adult animals in other studies probably belong to a reproducing pair, with high annual site fidelity, in contrast to raccoon dog in our study area) of the studied individuals, as well as our study area being the invasion front with low abundance of conspecifics. Even if the frequency of dispersal events and the turning angles did not vary seasonally among our nonreproducing individuals, we found indications that the step length of dispersing individuals was shorter in winter compared to the rest of the season. This is most likely related to that raccoon dogs adopt winter sleep during harsh climatic conditions, as well as snow conditions restricting movement (Kauhala et al. 2007; Zoller and Drygala 2013) and suggests a slower invasion during winter compared to the rest of the year under such climatic conditions. Note also that the number of observations per marked individual during winter months was rather low and may be biased toward periods where individuals were active and not in the winter sleep, which may affect our estimates of dispersal rate during winter.

The diurnal variation in movement pattern of raccoon dog appeared to follow the photoperiod, with longer steps and lower turning angles (more directional movement) in the twilight and dark hours (Fig. 2), supporting previous results about the nocturnal behavior of raccoon dog (Kauhala et al. 2007). However, the diurnal pattern in tortuosity was stronger for dispersing individuals. This was caused by lower turning angles (less tortuosity) during twilight and dark hours for dispersing individuals (Fig. 2E–G). Accordingly, the fine‐scale movement pattern of dispersing individuals differs from that of nondispersers mainly during dark hours. Moving into novel habitats can increase mortality, and raccoon dog most likely maximizes survival by restricting such movement to periods with less predation risk, which is assumed to be the dark hours (Kauhala et al. 2007). However, the movement pattern did not fully covary with the light regime; rather, it was characterized by long steps and more directional movement in the 2–3 h before and after dusk and dawn during spring and summer (Fig. 2). At high latitudes, such as our study area, animals may face no option than trading off risk avoidance (i.e., being active only during dark hours) with activities such as foraging, resting, and movement also in the light hours, simply because the night is too short (Swinnen et al. 2015). It should also be noted that the abundance of potential predators in the study area is low (e.g., Chapron et al. 2014), and of a total of more than 100 marked raccoon dog by the project, none was found killed by a predator (F. Dahl, pers. obs.). Accordingly, natural predation may be reduced as a factor shaping diurnal movement pattern. Instead, diurnal activity pattern may be shaped by avoiding periods where humans, also an important predator (Kowalczyk et al. 2009), are active (Lykkja et al. 2009). Nevertheless the reason for the diurnal pattern, the consequence is that movement related to dispersal occurs when the chances for humans to detect individuals are low due to low human activity.

A general pattern in all our results was the low degree of sex‐specific movement patterns. This contrasts the pattern found in many mammals (e.g., Greenwood 1980; Lawson Handley and Perrin 2007; but see Groó et al. 2013). Given the lack of sexual size dimorphism (Kauhala 1993; Melis et al. 2010) and that both males and females take part in raising the cubs (Kauhala et al. 1998), our result is not surprising and has also been reported for other studies on raccoon dog movement or habitat utilization (Kauhala et al. 1993; Drygala et al. 2008a; Melis et al. 2015).

Our simulation of encounter frequency and distance from source area to encounter sites showed as predicted; the lack of sex‐specific movement pattern gives a higher encounter rate and at larger distances compared to a scenario where males move faster than females. Our simulation is not an attempt to quantify with high accuracy the frequency and distance of encounter events; rather, it shows the relative difference in important determinants of invasion speed between realistic scenarios of sex‐specific movement pattern for mammals. Our simulation approach has some limitations. For instance, we did not consider habitat‐specific movement pattern (which could act as corridors for dispersal and thus increase encounter rate) or incorporating spatial autocorrelation in the movement (Fagan et al. 2013). Nevertheless, our results give a clear indication of how sex‐specific movement pattern can affect the speed of invasion. Specifically, the lack of sex‐specific movement patterns in raccoon dogs has consequences for both the frequency of male–female encounters (representing potential new settlements of breeding pairs) and the distance from the source population to the encounters (Table 3, Fig. 5). Invasive species disperse into areas uninhabited by the species and sex‐specific dispersal (either rate or distance) would give an invasion front consisting of one sex only, with no reproduction as the result. The similar dispersal behavior of male and female raccoon dog gives both a higher frequency of male–female encounters and larger encounter distances from the source population compared to the scenario with sex‐specific dispersal pattern. Combined with the fact that male and female raccoon dogs utilize similar habitats when on dispersal (Melis et al. 2015), the probability for male–female encounters, with potential for successful reproduction, at long distances from the source area is quite high. Similar mechanisms have been proposed as one of the explanations for the rapid expansion of roe deer (*Capreolus capreolus*) in Scandinavia (Andersen et al. 2004) and are probably also an important factor for the fast and successful colonization of raccoon dog in Europe (e.g., Helle and Kauhala 1991), together with factors such as a wide and omnivorous diet, a rather wide habitat utilization (Melis et al. 2015), and lack of natural predators such as lynx and wolf (Kowalczyk et al. 2009) in large parts of Europe (Chapron et al. 2014).

A consequence of the lack of sex‐specific dispersal patterns is that conservation efforts to find and eradicate dispersers should have highest priority. However, the lack of clear habitat associations in raccoon dog movement (Melis et al. 2015), that dispersal occurs throughout the year (Fig. 1) and the diurnal pattern in movement with most directional and fastest movement during night (Fig. 2) resulting in dark hours being the period to encounter dispersing individuals (Fig. 3), makes targeting such actions challenging.

## Conflict of Interest

None declared.

## Supporting information


**Data S1.** Evaluation of animal treatment effects on movement patterns.
**Figure S1.** Locations and home ranges (95% minimum convex polygon) for five raccoon dog pairs (red = females, blue = males).
**Figure S2.** Displacement (distance from the mean location of an individual at a given day, to the release location) in relation to days since release, for individuals that were released to the same area as they were captured (unmoved, blue points) and translocated individuals (red points).
