# Supplementary material for: Lack of sex‐specific movement patterns in an alien species at its invasion front – consequences for invasion speed
Source: Ecol Evol. 2016 Jul 14;6(16):5570–84. doi: 10.1002/ece3.2300 (PMC4983575; doi:10.1002/ece3.2300)
Supplement: Supplementary file 1 — Data S1. Evaluation of animal treatment effects on movement patterns. Figure S1. Locations and home ranges (95% minimum convex polygon) for five raccoon dog pairs (red = females, blue = males). Figure S2. Displacement (distance from the mean location of an individual at a given day, to the release location) in relation to days since release, for individuals that were released to the same area as they were captured (unmoved, blue points) and translocated individuals (red points). [file ECE3-6-5570-s001.docx]

**Supplementary material S1: Evaluation of animal treatment effects on movement patterns**

The individual raccoon dogs used in the study was marked with GPS-collars for two main purposes; 1) for management reasons, where marked animals were used to find other raccoon dogs, and 2) to acquire more knowledge about the spatial behaviour of raccoon dog at the invasion front. Particularly the first purpose implied some treatment of individuals that may potentially affect the behaviour, namely sterilisation and translocation.

Individuals were sterilised vasectomy for males and by tubal ligation for females before the release back in the nature. The sterilisation procedure retains all hormonal activity of the individual, but any mating attempt will be non-successful as the individuals are not fertile. Such sterilisation methods have been shown to not affect natural behaviour in other animals, except of course during the period of raising offspring for reproducing individuals. It is important to emphasize that the individuals are sterilized, but not neutered which would probably have affected their drift to find a partner. Accordingly, activities such as mate search, pair bounding, and mating, is expected to similar as for un-sterilised individuals, but there will be no cost of reproduction associated with producing and rearing offspring. Sterilisation was particularly important from a management perspective, in order to prevent reproduction in case a marked individual found a mate and established a pair after it was released. This could particularly be a problem in case of GPS-failure when it would be very hard to capture the marked animal or its mate to prevent reproduction. Raccoon dog are monogamous and form pairs that share the same territory until one dies. A sterilised raccoon dog will not raise offspring, and the lack of such a constraint on movement could potentially induce pair break-up or changing ranging behaviour (e.g. trigger dispersal). In order to investigate if our results could be affected by such behavioural responses to sterilisation, we took advantage of 5 know pairs among our GPS-marked individuals where we had location data for both the male and the female, starting the trajectories from both individuals in a pair on the first date with location from both the male and the female. We then used all locations until 10, 20, 30, 40, 50, 100 and 200 days after the start of the trajectories, and calculated 95% minimum convex polygon home ranges for male and female separately. Fig. S1 shows that the home ranges for male and female in a pair overlaps almost completely at every time steps and for all pairs. Moreover, the size the home ranges does not increase much after 30 days from first observation, which suggests that both the male and the female stays in confined home ranges with size of approximately 10 km^2^ (mean after 40 days: male = 10.2km^2^ ± 3.51 se, female = 9.9km^2^ ± 4.47 se, mean after 100 days; male = 12.0km^2^ ± 2.18 se, female = 12.4km^2^ ± 3.42 se). Accordingly, there is no evidence that sterilisation affects raccoon dog pair formation or the spatial behaviour of males and females in established pairs.

Some individuals were translocated to a different location from its capture location, which mean that it was introduced into a potentially novel environment. This was done in order to reveal if there were other raccoon dogs present in the new area. Such translocation may have implications on movement patterns, for instance by triggering long-distance movement such as dispersal. In order to investigate this further, we made a comparison of movement of individuals that were translocated and individuals that were released in the same area that they were captured in. All individuals had lost their partner before they were released, and their movement where therefore not affected by being in a pair. We then calculated the net displacement after 1, 5, 10, 20, 30, 40 and 50 days from release, as the distance from the location of release to the mean location at the time step in question. We then used standard t-tests to assess if the displacement (sqrt-transformed) differed between non-moved and translocated individuals, expecting a higher displacement of translocated individuals if the fact that they were introduced into a novel environment triggered dispersal-type behaviour. The tests gave no significant difference in the displacement between translocated and non-moved individuals at any time-step (Fig. S2). The non-significant longer displacement for translocated individuals was attributed to one single individual with a quite extreme movement, being more than 100 km away from the release location after only 20 days. However, such extreme movement was also present in one of the unmoved individuals, which started long-distance displacement after about three months from release, and then moved even further than the extreme translocated individual. Consequently, we have no evidence that different treatment resulted in individual variation in dispersal behaviour such as propensity to disperse or distance moved from the release location.


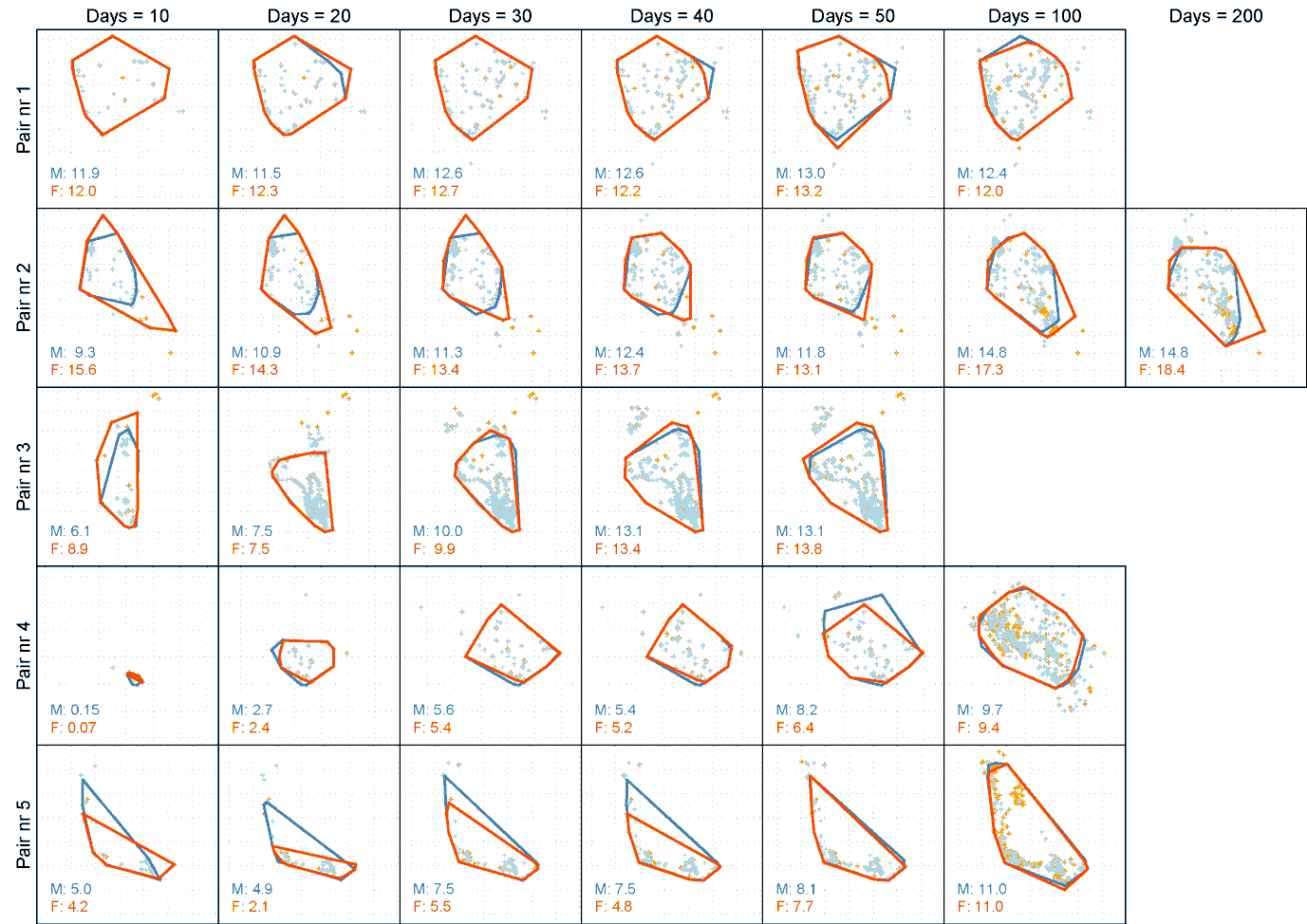


Fig. S1. Locations and home ranges (95% minimum convex polygon) for five raccoon dog pairs (red = females, blue = males). The first column shows locations and corresponding home ranges for the ten first days since the first date with location of both individuals in the pair, the second column for the first 20 days, etc. Each row shows one pair. Home range size (km^2^) for male (M) and female (F) is given in each panel. Missing panels mean that the pair was broken due to death of one or both individuals at that time.


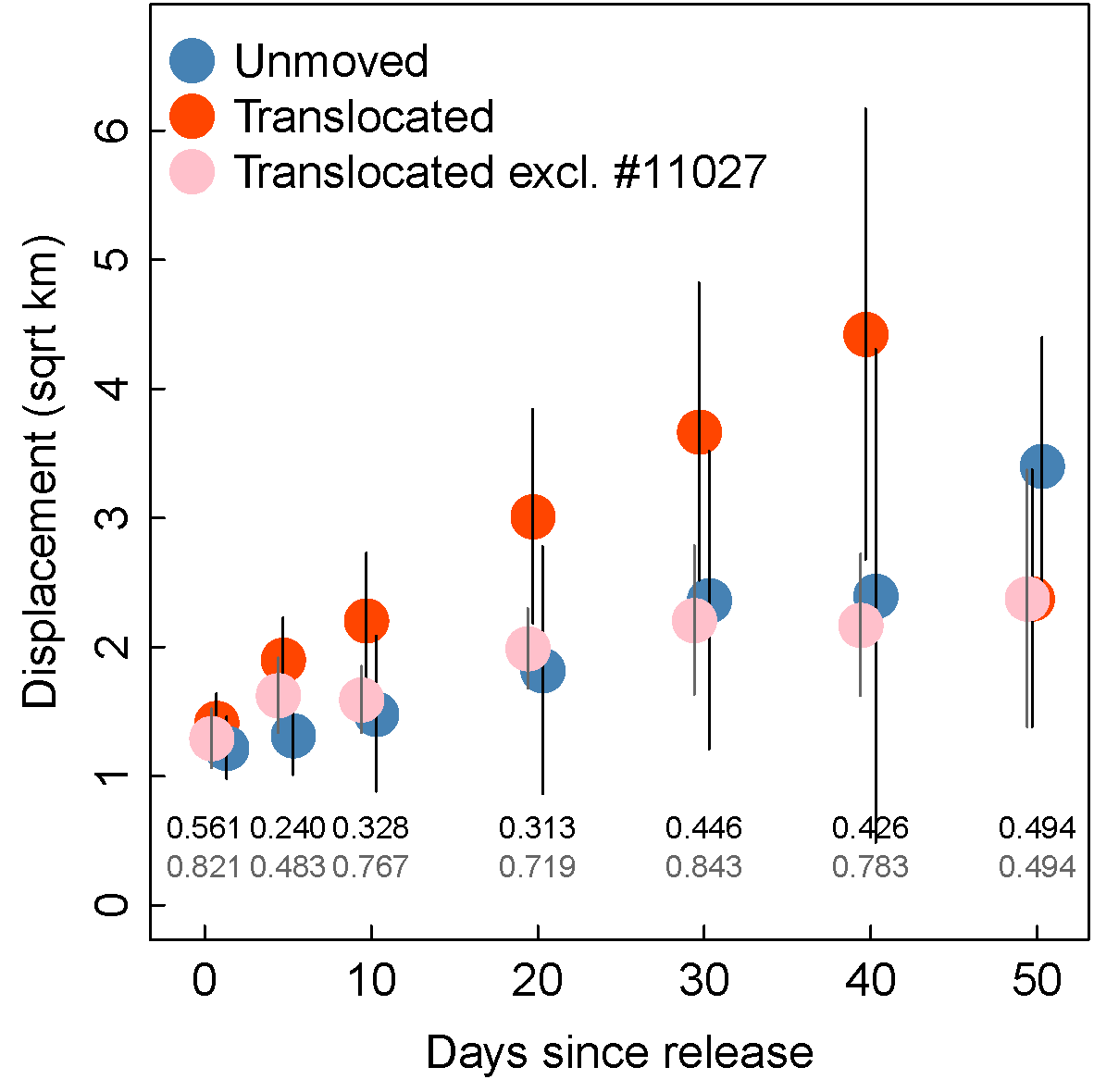


Fig. S2. Displacement (distance from the mean location of an individual at a given day, to the release location) in relation to days since release, for individuals that were released to the same area as they were captured (unmoved, blue points) and translocated individuals (red points). Bars show standard errors of the estimates. Light red points show translocated individuals excluding one individual that was > 100 km away from the release point already after 20 days. Values below the points show p-values from t-test of difference in the net displacement (sqrt-transformed) between the two groups, where black values are for all individuals and gray values for individuals excluding the one extreme individual. N = 17 release events (9 unmoved, 8 translocated), but decreasing with increasing day since release, and for 50 days since release the number of unmoved and translocated individuals is 5 and 5.
